# The Influence of *Contracaecum* larvae (Nematoda, Anisakidae) Parasitism on the Population of Prussian carp (*Carassius gibelio*) in Lake Sakadaš, Croatia

**DOI:** 10.3390/pathogens11050600

**Published:** 2022-05-21

**Authors:** Nera Vuić, Ivana Turković Čakalić, Barbara Vlaičević, Milica Stojković Piperac, Dubravka Čerba

**Affiliations:** 1 Department of Biology, Josip Juraj Strossmayer University of Osijek, Cara Hadrijana 8/A, HR-31000 Osijek, Croatia; nera.vuic@biologija.unios.hr (N.V.); ivana.turkovic@biologija.unios.hr (I.T.Č.); barbara.vlaicevic@biologija.unios.hr (B.V.); 2 Department of Biology and Ecology, Faculty of Science and Mathematics, University of Niš, Višegradska 33, 18000 Niš, Serbia; milicas@pmf.ni.ac.rs

**Keywords:** endoparasites, nematodes, freshwater fish, Fulton’s condition factor, negative allometric growth

## Abstract

*Contracaecum* larvae are geographically widely distributed, utilizing many animal species as hosts; and fish represent an important paratenic host in their life cycle. Their presence in Prussian carp (*Carassius gibelio*) was studied in Lake Sakadaš (Croatia) in 2017 and 2018. Two gill nets of different sizes submerged during a 12-h period were used to collect the fish. *Contracaecum* larvae were recorded in the stomach, slightly coiled or elongated on the intestine serosa or encapsulated in a gut wall of 20 individuals. The effect of *Contracaecum* sp. on the health of their host was determined by estimating the effect of the parasites’ presence, number, and biomass on fish length, weight, and the Fulton’s condition factor (CF). Infected fish showed negative (*b* < 3; *p* < 0.05) and uninfected fish positive allometric growth (*b* > 3; *p* < 0.05). Fish weight and CF in infected individuals were significantly low in comparison to the uninfected ones (Mann–Whitney *U* test: *U* = 1078.00, *U* = 423.50, respectively; *p* < 0.004). These results emphasize the importance of evaluating parasitic nematode presence in economically important fish species. Even more, if this endoparasite has a detectable negative impact on a resilient species such as the Prussian carp, it is important to monitor its occurrence in other fish species.

## 1. Introduction

According to the present knowledge, approximately 40% of all currently familiar animal species are classified as parasitic organisms that have successfully incorporated into ecosystems through complex interactions at all trophic level in different communities [1]. Such immense abundance and cosmopolitan distribution of parasitic species are result of their capability of exploiting most free-living organisms as their hosts, either intermediate, paratenic, or definitive [2]. Fish are very frequently infected by either endo- or ectoparasites, or both, and can have important role in their life cycle, often transmitting infection to other animals which feed on them, including humans [3]. Parasitic infection can cause a variety of pathological changes which can be quite detrimental to the fish health, including tissue damage, organ obstruction, and nutrient deprivation [4]. Consequently, infected fish may differ from the healthy fish, through the values of Fulton’s condition factor, length–weight relationship, indicating the severeness of infection. Changed values and ratios can indicate future disturbances in the growth rate and reproductive success [5,6].

Helminth parasites, generally comprised of five groups of invertebrates: Acanthocephala (spiny-headed worms), Trematoda (flatworms), Monogenea, Cestoda (tapeworms), and Nematoda (roundworms and nematodes), represent some of the most common metazoan endoparasites found living obligately inside various organs of fish [7]. According to Anderson [8], of about 2272 described genera of Nematoda, at least 33% are well-known parasites in vertebrates. Specifically in fish, genera *Anisakis*, *Contracaecum*, *Hysterothylacium*, and *Pseudoterranova* are regarded as some of the most prominent nematode parasites, especially concerning their zoonotic potential [4]. Most of the aforementioned genera belong to the family Anisakidae, except the genus *Hysterothylacium* which is included in the family Raphidascarididae [9,10]. According to Shamsi [11] the genus *Contracaecum* is known as the most species diverse genus in the family, containing more than 100 species; however, most recent information from the World database of Nematodes [12] lists a total of 39 confirmed species in this genus. *Contracaecum* is geographically widely distributed, successfully utilizing a vast variety of invertebrate and vertebrate species as hosts, both terrestrial and aquatic organisms [11,13].

In the complex life cycle of species within the genus *Contracaecum*, eggs get into the water through the feces of the final host, a piscivorous bird or marine mammal [11]. In the water, eggs and larvae can be eaten by aquatic arthropods or other invertebrates that serve as first intermediate host [14,15,16]. Fish are second hosts, commonly known to harbor third stage larvae, usually located in the intestinal wall and the mesentery, or inside muscles or liver [11,17,18]. If no further development of the parasite occurs within the fish, they are referred to as paratenic hosts. This specific kind of host is not necessarily needed for a successful completion of the life cycle, but serves as an additional source of infection for other potential hosts [19,20,21]. Some of the most common fish hosts of *Contracaecum* in freshwater systems include cyprinids, ictalurids, and cichlids, while whiting, capelin, and cod represent the most common fish hosts in marine systems [20]. The *Contracaecum* life cycle can be completed if the infected fish is eaten by an appropriate definitive host associated with aquatic environment (e.g., cormorants) [22].

Accidental infection of humans with *Contracaecum* third stage larvae is possible by consuming the raw, undercooked, or, in other ways, inadequately prepared fish [23,24], although infections by members of the *Anisakis* genus are far more prominent [25]. These infections are characterized as a zoonotic disease called anisakiasis or anisakidosis [26]. Depending on a number of factors, such as the location of the parasites, their subsequent migration through the body, and host’s immune response, anisakidosis can be presented as either intestinal, gastric, or allergic, with different symptoms varying from mild to severe [27,28,29]. Reports of such infections have been increasing in frequency [25,30], most likely as a result of the increasing consumption of raw and undercooked fish around the world [31,32], as well as Croatia [33].

Prussian carp (*Carassius gibelio* (Bloch, 1782)), is a cyprinid fish species, highly invasive in non-native rivers, floodplains, and standing waters, whose opportunistic lifestyle, diverse diet, gynogenetic reproduction, and high tolerance to a wide spectrum of living conditions have enabled its successful spreading throughout the Europe as well as the rest of the world [34,35,36]. This species has established stable populations in a variety of freshwater ecosystems, where it acts as a strong competitor to native fish. For some species, such as common carp (*Cyprinus carpio*), Prussian carp is considered to be one the main causes of its endangerment and a substantial decrease of abundance [37]. Such characteristics also make it an ideal candidate for the infection and subsequent spread of various endoparasitic species, as observed with findings of Protozoa [38], Monogenea [39], Digenea [35], Trematoda [35], Acanthocephala [40], and Nematoda [4], parasites in Prussian carp, including the genus *Contracaecum* [41]. The individuals of Prussian carp, infected by the third stage of *Contracaecum* larvae, could show high zoonotic potential due to their frequent use in recreational and sport fishing, as well as a source of food for humans, making it extremely important to monitor their health [34].

Prussian carp was found to be highly abundant in the ichthyocenoses of Kopački Rit floodplain [42]. Different water bodies of the floodplain represent suitable habitats for living, eating, and spawning of many fish species, sustaining diverse communities and food sources. Correspondingly, many piscivorous predators can be found, such as pike, otters, cormorants, eagles, and other fish-eating birds [42,43,44,45]. Even though it is prohibited to catch fish in most parts of the Kopački Rit Nature Park, fishing is allowed in the water bodies surrounding the area, and the Danube and Drava River what represents potential way of human infection in this area. In addition to its status as a Nature Park on a national level, Kopački Rit is also internationally recognized as an Important Bird Area and is included in the Ramsar list [46]. High biological productivity makes this natural floodplain an important spawning area for various fish species of Drava and Danube rivers. Out of 44 documented fish species, 35 are native, while 9 are considered introduced [47]. Additional research indicates a record of 50 fish species in the Nature Park [48]. Kopački Rit is an important resting and nesting area for numerous and diverse ornithofauna consisting of more than 300 bird species [47,49,50], including large colonies of cormorants *Phalacrocorax carbo*, a common final host in the *Contracaecum* life cycle [8,16,51].

Therefore, the aim of this study was to (1) validate and analyze the parasite infection present in Prussian carp from a floodplain lake; (2) determine the intensity of parasite infection and establish its correlation to fish morphometric parameters; and to (3) verify possible negative effects of parasites on fish health by evaluating variances in Fulton’s condition factor and length–weight relationships between healthy and infected individuals.

## 2. Results

**Figure 1:**
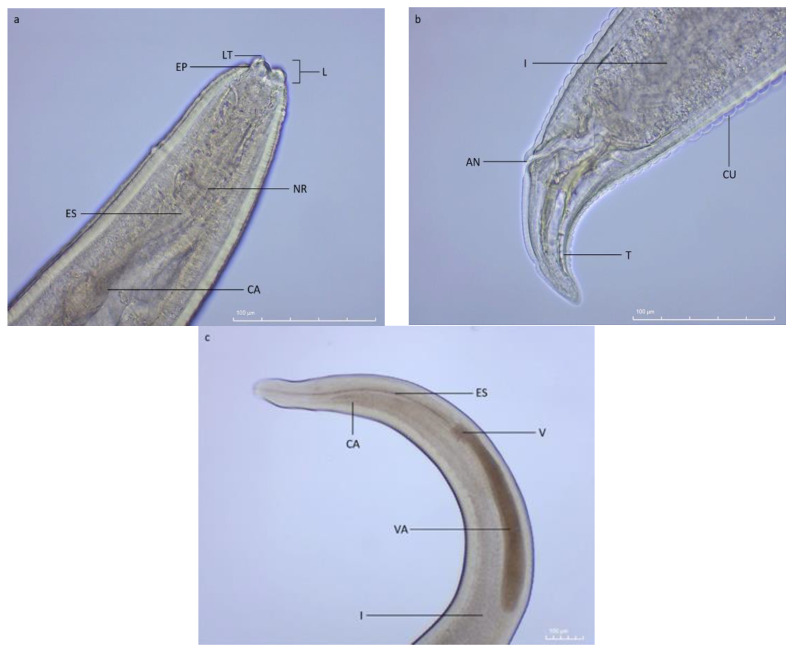
Lateral view of the third stage *Contracaecum* sp. larvae isolated from the stomach of Prussian carp (*Carassius gibelio*): (**a**) anterior end: L—underdeveloped lips, LT—larval (cephalic) tooth, EP—excretory pore, NR—nerve ring, ES—esophagus, CA—intestinal caecum; (**b**) posterior end: I—intestine, AN—anus, CU—striated cuticle, T—tail; (**c**) anterior end: CA—intestinal caecum, ES—esophagus, V—ventriculus, VA—ventricular appendix, I—intestine.

A total of 199 collected individuals of Prussian carp (*Carassius gibelio*) were examined. None of them harbored any macroscopic ectoparasites; however, 20 individuals were infected with the nematode endoparasite identified as *Contracaecum* sp. third stage larvae, Type II, based on the main morphological characteristics (Figure 1).

The comparison of morphometric parameters between infected and uninfected individuals of Prussian carp is given in Table 1. Out of 20 infected individuals, 7 were males and 13 were females. Comparison of differences between sexes in fish morphometric parameters, Fulton’s condition factor and parasite intensity is given in Table 2.

**Figure 2:**
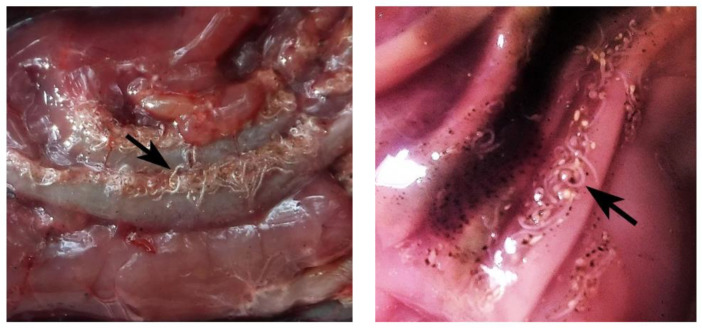
*Contracaecum* sp. larvae (arrow) in the stomach of Prussian carp (*Carassius gibelio*).

The third stage larvae (L3) of *Contracaecum* sp. were found in the stomach of Prussian carp (Figure 2), slightly coiled or elongated, on the serosa of the intestine or encapsulated in gut wall. The parasite prevalence was 10.1%. Intensity of infection varied considerably, with the minimum number of nematodes being 7, and the maximum 1078, giving a mean intensity of 366 nematodes per infected fish. The variance was 21,930, while the variance to mean ratio was 597. Mean abundance was 36.7. Average nematode total biomass was 33,245.5 µg, with the minimum of 307.9 µg and a maximum of 141,660 µg per infected fish.

Calculations of length–weight relationships showed differences in growth between infected and uninfected fish, evident in coefficient *b* values, with infected fish showing negative allometric growth (*b* = 2.00; H_0_ : *b* = 3 rejected at the 0.05 significance level), and uninfected fish positive allometric growth (*b* = 3.32; H_0_ : *b* = 3 rejected at the 0.05 significance level). The length–weight relationship for infected fish was established through the equation $W=0{.60\;\times \;L}^{2.00}$ (R^2^ = 0.70), and $W=0{.01\;\times \;L}^{3.32}$ (R^2^ = 0.87) for uninfected fish.

Fish weight and Fulton’s condition factor in infected individuals were significantly low in comparison to the uninfected ones (Mann–Whitney *U* test: *U* = 1078.00, *p* = 0.004; *U* = 423.50, *p* = 0.000, respectively). In contrast, total body length showed significantly higher values in infected specimens (Mann–Whitney *U* test: *U* = 1054.00, *p* = 0.003). No difference in standard length between infected and uninfected individuals was observed (Mann–Whitney *U* test: *U* = 1750.00, *p* = 0.870). The high standard deviation for each measured fish body parameter of uninfected individuals indicates that the data are more spread out around the mean. On the other hand, considering the differences of all measured parameters between sexes (Table 2), only fish weight significantly differed between infected males and females (Mann–Whitney *U* test: *U* = 17.00, *p* = 0.024). In addition, Spearman’s correlation coefficient revealed significant negative correlation between CF and each of the three parasite infection parameters (Table 3).

## 3. Discussion

Larval stages of *Contracaecum* (Railliet and Henry, 1912) are known to infect various species of freshwater fish, especially those in the Cyprinidae family [18]. In this research, Prussian carp (*Carassius gibelio* (Bloch, 1782)) adult individuals were infected with the Type II third stage larvae (L3) of *Contracaecum* nematode (Nematoda: Anisakidae). Curiously, no other taxa of macroscopic ectoparasites or macroscopic helminth endoparasites were discovered in the Prussian carp. In comparison to the research of Demir and Karakişi [39], Daghigh Roohi et al. [38] and Pakosta et al. [52], who discovered a total of three, four and eight different taxonomic groups of parasites respectively, the diversity of parasitofauna of Prussian carp in this research was considerably low.

The larvae were found inside serosa layer of the gastrointestinal wall of the fish, coiled and surrounded by a layer of connective tissue, completely inactive but with no visible signs of decomposition. Similar findings of *Contracaecum* larvae were reported in bream (*Abramis brama*) and common carp (*Cyprinus carpio*) [18] and experimentally infected asps (*Leuciscus aspius*) [53]. *Contracaecum* larvae have the tendency to migrate from the intestine to surrounding host’s organs by burrowing through tissue, causing mechanical damage along the way and eliciting an immune response [54,55]. Therefore, the tissue encapsulating the nematodes found in Prussian carp could presumably be a sign of a former inflammatory reaction triggered at the earliest stages of the infection, most likely during the aforementioned migration of larvae.

Prevalence of *Contracaecum* sp. parasites observed in this study is relatively low compared to findings described in Boane et al. [56] for *Cyprinus carpio* (17.4%) and Younis et al. [57] for *Hydrocynus forskahlii* (82%) and *Lates niloticus* (100%). The aforementioned two studies, including this one, relied on visual examination as a method of parasite detection in host tissue. However, it is important to note that additional inspection and isolation using the pepsin digestion [58] or room temperature incubation method [59] or UV-light method [60] could give more precise results regarding parasite prevalence and intensity of infection. Compared to the standard visual inspection, Shamsi and Suthar [59] recorded higher values of prevalence, mean abundance and mean intensity using the incubation method. Therefore, a possibility exists that the values of parasite infection indices in this research could have been even higher if an additional parasite isolation method was applied.

Even though the value of mean intensity was quite high (366) compared to Demir and Karakişi [39] (1.40), the values varied considerably between infected fish, ranging from just 7, to a maximum of 1078 nematodes. This is a common occurrence in macroparasite infections [61] and depends on various factors, such as host susceptibility, behavior, host population size, etc. [62,63].

Fulton’s condition factor and length–weight relationships are some of the most commonly used parameters in fishery research and management [64], offering information about the general condition, fatness, growth rate, and age structure of fish populations [65,66,67]. In parasitological research, these parameters can be used to describe effects of parasite infections on host health [68]. Infected individuals of Prussian carp in this study showed significantly lower values of Fulton’s condition factor compared to uninfected individuals, along with a negative correlation between the fish condition factor and parasite burden. These results correspond with recorded values of length-weight relationships, showing negative allometric growth (*b* < 3) for infected, and positive allometric growth (*b* > 3) for uninfected fish. Additionally, infected Prussian carp individuals showed significantly higher total length values opposed to the uninfected individuals, further corroborating the difference in growth rate between the two sample groups. According to Poulin [69], parasite burden tends to increase with the total length of fish host, which is known to be directly related to the age of the host. As older fish grow in size, they are able to accumulate more parasites throughout their life [70]. Al-Zubaidy [13] also detected positive correlation between the length of fish host (*Liza abu*) and parasite presence (*Contracaecum* sp.).

Even though the uninfected Prussian carp in this study had higher mean condition factor values compared to those recorded in Croatia (2.08 ± 0.34 and 1.79, respectively) [71], the infected Prussian carp had a mean value lower than both of the aforementioned (1.64 ± 0.14). Similarly, *b* coefficient value for length-weight relationships in infected fish was also lower (2.00) compared to both the healthy (3.32) and the mean *b* value of the Prussian carp populations in Croatia (3.29) [72]. Fish biometrics, values of Fulton’s condition factor, and length–weight relationships can also be influenced by other biotic and abiotic factors, such as the habitat, season, population density of fish, and food availability [73]. Since condition factor assumes heavier fish are in a better condition [73,74], results obtained in this research suggest that presence of *Contracaecum* larvae has detrimental effects on health of the infected Prussian carp. Similar findings have been reported in *Hoplias malabaricus* that were infected with *Contracaecum* parasites [5]. On the other hand, *Clarias gariepinus* shows resistance to adverse effects of *Contracaecum* infection [75] and did not show lower values of mean condition factor in infected individuals [17].

Third stage *Contracaecum* larvae do not seem to cause significant pathological changes in the Prussian carp, apart from a mild local tissue inflammation, as observed in this study and additionally by İnnal et al. [41]. However, it is possible for pathological changes to vary in intensity between host types, oftentimes becoming more severe in final hosts, as evident in [76,77] where the parasitic infection caused gastric ulcerations in cormorants and seals, respectively.

Given that and the results of our research, we emphasized the importance of the evaluation of parasitic nematode fauna presence in commercially important fish species. Even more, if this nematode has a detectable negative impact on a resilient species as the Prussian carp, it is important to monitor its occurrence in other fish species. To create a wider perspective on the presence and spread of the *Contracaecum* larvae in ichthyofauna of this area, sampling of the fish followed by the parasite inspection, should be conducted on more sites and during different seasons, with the thorough examination of fish musculature to assess the exact zoonotic potential. Furthermore, a molecular analysis of parasites to the species level is vital to further extend our knowledge on studied endoparasites.

## 4. Materials and Methods

### 4.1. Study Area

**Figure 3:**
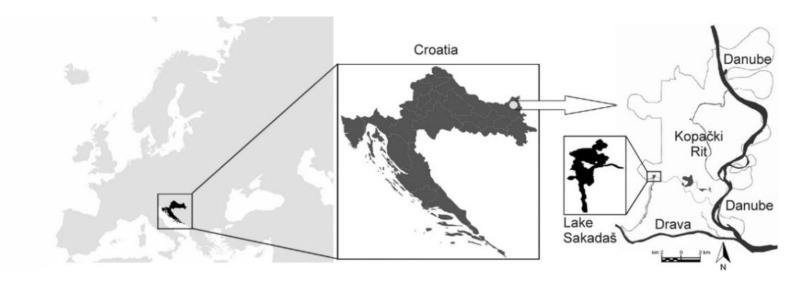
Position of the Lake Sakadaš within the Kopački Rit Nature Park, Croatia.

Located in the north-eastern part of Croatia, within the lowest part of the Baranja region, Kopački Rit is characterized as an inland delta formed at the confluence of Drava and Danube rivers. The investigated Lake Sakadaš is situated in the south-western part of the floodplain declared as Special Zoological Reserve (Figure 3). With a maximum depth of 12 m (average of 4–5 m), it is considered the deepest water depression in the area. This floodplain lake is connected to the Danube River by two channels, Hulovo and Čonakut [47,78].

### 4.2. Prussian carp (Carassius gibelio) Sampling and Parasite Inspection

Sampling of the Prussian carp (*Carassius gibelio*) was conducted in Lake Sakadaš, in the late autumn of 2017 and early autumn of 2018. Two gill nets of different sizes were used to capture the fish, one 20 m long with 14 mm mesh size, and other 80 m long with 16 mm mesh size. Both nets were placed in the lake at specific sites and left overnight for a 12-h period. The following morning all of the collected fish were examined. Each individual was firstly removed from the net, followed by the external inspection for macroscopic ectoparasites, which included thorough inspection of nostrils, eyes, gills, fins, scales, and underneath the operculum [79].

Total length (TL) and standard length (SL) of each fish was measured [80] using ichthyometer. Additionally, fish were weighed using a digital scale.

In the field, fish were sacrificed and a necropsy was performed starting with incisions along the ventral length, subsequently followed by incisions on the lateral sides leading dorsally. Once opened, the body cavity of the fish, along with internal organs and musculature, was examined for macroscopic endoparasites (helminths) and cysts. Infected fish were placed in containers and transported in a portable cooler with ice to the laboratory where they were immediately frozen. Smaller fish and those with suspected infection were also brought to the laboratory in a portable cooler with ice for additional, more precise inspection.

In the laboratory, all organs in the fish body cavity were separated from it, placed in a Petri-dish with distilled water and examined under Olympus SZX9 stereomicroscope (Olympus Optical Co. (Europa) GmbH, Hamburg, Germany). Endoparasites (nematodes) were carefully removed with fine forceps and preserved in 70% ethanol.

### 4.3. Parasite Identification and Measurements

Preceding slide preparation, nematodes were cleared in a glycerin and ethanol solution, and then mounted in glycerin [81]. From every infected fish sample, a total of 100 nematodes were randomly selected for slide preparation; if the number of nematodes did not exceed 100, all of the isolated nematodes were taken [82]. Nematodes were identified based on their morphological features under the Olympus BX51 microscope (Olympus Optical Co. (Europa) GmbH, Hamburg, Germany) using the following keys: Anderson et al. [83], Anderson [8], and Kanarek and Bohdanowicz [51]. Some of the main morphological characteristics used for the identification of nematode larvae included the presence of a larval (cephalic) tooth located in-between two, out of a total of three, underdeveloped lips (two ventrolateral and one dorsal lip), along with an excretory pore situated at the base of the lips. Gastrointestinal elements included a short ventriculus, followed by a longer ventricular appendix and an intestinal caecum located anteriorly, ending proximately to the nerve ring [5,84]. Similar characteristics, including length and width of the larvae, the distance between anus and the tail tip (including tail tip morphology), and the position of the nerve ring, were used to determine the type of larvae, according to Cannon [85]. Morphological features most important for the identification process have been presented and labeled on Figure 1, taken using the Motic Moticam 5 camera (Motic Group Co., Ltd, Wetzlar, Germany) and the Motic BA310 microscope (Motic Group Co., Ltd, Wetzlar, Germany). Nematode individuals were photographed with Olympus Camedia C-4040 camera (Olympus Optical Co. (Europa) GmbH, Hamburg, Germany) and measured using Olympus DP-Soft software (Olympus Optical Co. (Europa) GmbH, Hamburg, Germany) to calculate their biomass.

Measurements of nematode length and width were used to calculate the total nematode biomass (WW) per infected fish, using the Andrassy formula [86]:${\;\mathrm{WW}=L\;\times \;W}^{2}/1,600,000$, where L represents the nematode’s length (µm) and W the width (µm). Biomass was expressed in µg [82].

### 4.4. Parasite-Host Relationship

The effect of *Contracaecum* sp. larvae on the health of their host, the Prussian carp, was determined by estimating the effect of the parasites’ presence, number, and biomass on fish length, weight, and the Fulton’s condition factor. Fulton’s condition factor (CF) for each individual fish was calculated as $\mathrm{CF}=(W\;\times \;100)\;/\;{\mathrm{TL}}^{3}$, where W represents fish weight (g), and TL total body length [71,87].

In order to estimate the relationship between total length and weight of infected and non-infected fish, the length–weight equation ${W=\mathrm{aL}}^{b}$ was used [88], where a represents the intercept, b the slope of the relationship, W is the weight (g), and L the total length of the fish in cm. The parameters *a* and *b* were calculated using the linear regression of the log-transformed length–weight equation: log (W)=log(a)+b log(L) [66]. Furthermore, 95% CI-s were used to test for isometric or positive/negative allometric growth in infected and healthy fish, where the isometry null hypothesis (H_0_ : *b* = 3) was rejected at 5% significance level [65]. Analyses were performed in R using the package *stats* version 4.0.2 (R Core Team, 2020, Auckland, New Zealand) [89].

Parasitic infections are often characterized by an aggregated distribution of parasites in the host population, meaning that just a few hosts in the population will bear high numbers of parasites, while the rest will have very little to none. To properly characterize the parasite infection in collected Prussian carp, the Quantitative Parasitology 3.0 software was used to calculate the 95% CI for prevalence (Clopper-Pearson), mean intensity, mean abundance (BCa method with 2000 bootstrap replications), and variance to mean ratio [90,91].

### 4.5. Data Analysis

Since the normality assumption for the values of analyzed parameters was failed, the non-parametric alternatives of statistical tests were used. Non-parametric Mann–Whitney *U* test for two independent samples was used for testing the effects of parasites on parameters that reflect health of fish population. Moreover, Mann–Whitney *U* test was used to analyze the differences in fish morphometric parameters, Fulton’s condition factor, and parasite intensity between fish sex.

To obtain more compressive insight into the visualized relationships between variables, Spearman’s correlation coefficient was calculated to determine the relationship between the extent of parasite infection and the body condition of the host.

All analyses were performed using SPSS version 15.0 software (SPSS Inc., Chicago, IL, USA).

## 5. Conclusions

Our research presents first findings of nematode endoparasites *Contracaecum* and their relationship with freshwater fish Prussian carp (*Carassius gibelio*), in Kopački Rit Nature Park, Croatia. The negative influence of nematode parasites on invasive fish-species was evident in the changes of the growth capacity, i.e., lack of allometric growth pattern. The present study is a stepping stone for further extended research which would include molecular analysis, and, additionally, the monitoring of *Contracaecum* occurrence in other fish species. In this way, it would be possible to study infection and disease risks of native communities and the possibility of parasite “spill-over” or “spill-back” occurrences and effects on ichthyocenosis in this floodplain area. This would allow us to acquire an insight into complex parasite–host interrelationships and attempt to assess the influence on other organisms, including humans. Even though there are many unknown facts, it is important to raise this question if we are to manage biotic and abiotic resources in protected aquatic systems.

## Figures and Tables

**Table 1:** Host parameters of *Contracaecum*-infected and uninfected Prussian carp (*Carassius gibelio*) from Lake Sakadaš (Croatia).

Parameters	Infected Fish ^a^ (N = 20)	Uninfected Fish ^a ^ (N = 179)
TL ± SD (range)	36.5 ± 2.0 (33.2–44.4)	32.8 ± 7.6 (7.5–42.0)
SL ± SD (range)	28.6 ± 1.8 (26.0–32.5)	26.7 ± 6.3 (5.5–34.0)
Fish weight ± SD (range)	803.0 ± 105.4 (660.0–1053.0)	852.6 ± 324.8 (5.0–1410.0)
CF ± SD (range)	1.64 ± 0.14 (1.32–1.93)	2.08 ± 0.34 (0.98–3.02)

^a^ Mean values ± standard deviation (SD) for total length in cm (TL), standard length in cm (SL), fish weight in g, and Fulton’s condition factor (CF). N = number of fish.

**Table 2:** Mean values ± standard deviation (SD) for standard length in cm (SL), total length in cm (TL), fish weight in g, intensity (number of *Contracaecum* sp. per fish), and Fulton’s condition factor (CF) for infected male (M) and female (F) specimens of Prussian carp (*Carassius gibelio*).

Sex	SL ± SD	TL ± SD	Fish Weight ± SD	Intensity ± SD	CF ± SD
M (N = 7)	36.1 ± 1.4	28.4 ± 1.2	741.0 ± 51.0	414.1 ± 339.3	1.58 ± 0.15
F (N = 13)	36.7 ± 2.2	28.8 ± 2.1	836.0 ± 114.0	339.3 ± 319.2	1.68 ± 0.13

**Table 3:** Correlation between the total length (TL), standard length (SL), fish weight, and Fulton’s condition factor (CF), and the presence (PresAbsN), number (NoN), and biomass (massN) of *Contracaecum* larvae.

	TL	SL	Fish Weight	CF
PresAbsN	0.16 *	0.09	−0.05	−0.37 **
NoN	0.12	0.07	−0.03	−0.27 **
massN	0.09	0.05	−0.01	−0.19 **

Significant correlations are given at * *p* < 0.05 and ** *p* < 0.01.

## Data Availability

The data presented in this study are available on request from the corresponding author.
